# Gender differences in the perceived need for community-wide deworming: Formative qualitative research from the DeWorm3 study, India

**DOI:** 10.1371/journal.pntd.0008829

**Published:** 2020-11-25

**Authors:** Kumudha Aruldas, Arianna Rubin Means, Angelin Titus, Yesudoss Jacob, Rajeshkumar Rajendiran, Jabaselvi Johnson, Mira Emmanuel-Fabula, Saravanakumar Puthupalayam Kaliappan, Sanjay Kamlakar Juvekar, Gagandeep Kang, Judd L. Walson, Sitara Swarna Rao Ajjampur

**Affiliations:** 1 The Wellcome Trust Research Laboratory, Division of Gastrointestinal Sciences, Christian Medical College, Vellore, India; 2 Department of Global Health, University of Washington, Seattle, United States of America; 3 Vadu Rural Health Program, KEM Hospital Research Centre, Pune, India; 4 Department of Global Health, Medicine, Pediatrics & Epidemiology, University of Washington, Seattle, United States of America; 5 DeWorm3, Division of Life Sciences, Natural History Museum, London, United Kingdom; University of Cambridge, UNITED KINGDOM

## Abstract

Current soil-transmitted helminth (STH) programs target morbidity control with school-based deworming. Increasing interest in steering neglected tropical disease (NTD) programmes from morbidity control towards disease elimination has prompted evaluation of strategies that may interrupt transmission. The feasibility of interrupting transmission of STH with community-wide deworming is being tested in the ongoing DeWorm3 cluster randomized trial. Gender-based perspectives about susceptibility to infection and need for treatment have been shown to influence both health-seeking behaviour and health outcomes. We carried out a qualitative study among men and women in the community to understand their knowledge, beliefs, and attitudes about STH infections and community-wide mass drug administration (cMDA). Eight semi-structured focus group discussions were conducted among men and women residing in the DeWorm3 study site in India—Vellore and Tiruvannamalai districts of Tamil Nadu. Thematic coding was used to analyse the transcripts in ATLAS.ti 8.0. Both men and women in this study demonstrated a high level of STH knowledge but some men had misconceptions that intestinal worms were beneficial. Men and women shared several similar beliefs and attitudes regarding STH treatment. Both believed that adults were likely to have STH infections and both reported that stigma prevented them from seeking treatment. Influenced by gender norms, women were more likely to associate STH infections with inadequate sanitation and hygiene, while men were more likely to believe that those engaged in agricultural work were at risk. Both genders reported a positive attitude towards cMDA for STH. Barriers to cMDA implementation differed by gender; women expressed concern regarding side-effects and drug quality while men were concerned that treatment coverage may be affected due to the absence of people during the day when the drug is distributed. Both men and women perceived the treatment of adults for STH infections to be important, however, the perceived barriers to participating in cMDA differed by gender in this community. The study identified key messages to be incorporated in communication and outreach strategies for cMDA programmes.

## Author summary

In this qualitative study, we conducted focus group discussions with men and women residing in communities participating in a cluster-randomized trial of community-wide deworming for soil-transmitted helminth (STH) in order to understand how knowledge, beliefs, and attitudes are influenced by gender. Men and women shared similar beliefs and attitudes, although there were differences in knowledge about STH infection and its treatment. While both genders understood that STH transmission is associated with poor sanitation and hygiene, women voiced a greater appreciation of these risk factors, perhaps because of the STH knowledge gained from the frontline health workers during school MDA and anaemia programs. Men and women both believed that adults may be re-infecting children in the community and both articulated acceptance of deworming medications. However, both men and women also noted that stigma associated with an adult having STH infection prevented them from actively seeking treatment. The cMDA for STH, may provide important socio-behavioural benefits to communities, as it provides an opportunity to treat all community members over one year of age without discrimination by infection status or age. However, compliance with treatment may be affected by community beliefs; women expressed concerns regarding treatment safety while men specifically expressed concerns about achieving treatment coverage due to the absence of some individuals during cMDA due to migration and people being absent for work. Findings from this study suggest that communication and dissemination messages should include a rationale for cMDA and stress the safety and benefits of the drug. In addition, these results highlight the importance of carefully timing the delivery of cMDA to ensure that working adults and migratory populations have access.

## Introduction

India accounts for 21% of the 1.45 billion people estimated to be infected with soil-transmitted helminths (STH) globally [1]. The World Health Organization’s (WHO) target for endemic countries is to reach 75% of at-risk pre-school aged children (PSAC) aged (1–4 years) and school-aged children (SAC) aged (5–14 years) with deworming treatment by 2020 [2]. Based on these recommendations, the Ministry of Health & Family Welfare (MOH&FW), Government of India, initiated the National Deworming Day (NDD) programme in 2015 where all PSAC and SAC are targeted for deworming biannually in endemic areas [3]. In one of the largest mass drug administration (MDA) programs in the world, an estimated 226 million children were treated by the NDD programme in 2018 [4]. This is a multi-sectoral initiative where, in addition to MOHFW, implementation takes place through the combined efforts of Department of School Education and Literacy under Ministry of Human Resource and Development, Ministry of Women and Child Development and Ministry of Drinking Water and Sanitation.

Recent mathematical modelling studies have indicated that it may be possible to interrupt the transmission of STH by expanding treatment eligibility to individuals of all ages [5,6]. The DeWorm3 study is a multi-country community cluster randomized community-based trial being conducted in Benin, India, and Malawi testing the feasibility of interrupting STH transmission using community-wide MDA (cMDA) compared to standard of care school-based deworming programmes [7]. This hybrid implementation-effectiveness trial also uses mixed-methods to optimize the delivery of trial interventions and generate potential recommendations for translation of study evidence into relevant changes in policy and practice [8]. At study baseline, prior to the launch of the DeWorm3 intervention, qualitative formative research was conducted among men and women residing in these communities to identify barriers and facilitators to cMDA and opportunities for streamlining and optimizing community wide STH programmes.

Evidence suggests that gendered beliefs and norms influence healthy practices and health-seeking behaviour and may contribute to disparities in health outcomes resulting from neglected tropical diseases (NTDs) (including STH), diarrhoeal diseases, HIV and other maternal and child health conditions [9–13]. A recent systematic review and meta-analysis demonstrated that the prevalence of *Schistosoma japonicum* and hookworm was higher among males reflecting the role of gender norms like fishing and farming to be the reason for higher prevalence among men [11]. While one analysis of MDA coverage from 16 countries found that MDA coverage is generally gender equitable [12], in 2016, the median coverage for all diseases was slightly higher for women than men. For lymphatic filariasis (LF), only two countries, Haiti and Indonesia, reported higher coverage among men than women. Studies of MDA for lymphatic filariasis (LF) in India also observed discrepancies in MDA compliance (proportion of people who take a drug after receiving it) by gender in different regions; men had higher treatment compliance than women in Odisha but in Maharashtra, coverage was lower among men [14,15]. Deliberate examination of gender equality and equity in health research and health programmes may be one strategy for reducing these disparities [16]. However, there are few studies on the gender-based factors that influence participation in MDA programs, particularly in the Indian sub-continent, where the largest proportion of the world’s population at-risk of STH resides.

We describe gender-based perspectives of STH infection and cMDA for STH among adult men and women residing in the community in India where the DeWorm3 trial is being conducted. We investigated community members’ beliefs and knowledge of STH, perceived effect of STH treatment on health and wellbeing, and attitudes toward the cMDA programme.

## Methods

### Ethics statement

The study was approved by the Institutional Review Board of Christian Medical College, Vellore (10392 [INTERVEN]) and the Human Subjects Division at the University of Washington (STUDY00000180). Written consent was obtained from each individual participating in the FGDs.

### Study location and setting

The DeWorm3 study site in India includes Timiri block in Vellore district and Jawadhu hills block in Tiruvannamalai district, both located in the southern state of Tamil Nadu. The detailed study protocol of the parent trial has been published elsewhere [7,8]. Briefly, the feasibility of interrupting transmission of STH is being evaluated by comparing the effect of three rounds of biannual community-wide deworming to standard of care biannual school-based deworming on STH prevalence. Concurrently, the study is also collecting data to inform opportunities, challenges, and best practices for implementing future STH transmission interruption programs. The study covers an enumerated population of 140,932, both rural and tribal, residing in 36,536 households, divided into 20 intervention and 20 control clusters.

### Data collection

Qualitative data were collected with community members in four randomly selected trial intervention clusters in February 2018, as part of a formative study before any community sensitisation activities began and cMDA was delivered. The FGDs were conducted in four intervention clusters to capture potential variations in prior cMDA experiences across geographic areas that may influence current perceptions of cMDA. In these communities, we conducted eight focus group discussions (FGDs), four with adult men and four with adult women to identify factors influencing perceived barriers and facilitators to participating in cMDA. As these FGDs were conducted at the baseline of the DeWorm3 trial prior to the collection of any individual-level prevalence data, purposive sampling was used. A quota of 20 men and women (a minimum of five women or men per FGD in each of the four clusters) were included, allowing only one willing adult man or woman from a household having no leadership position in the communities to participate. Considering the cultural context where women would not sit among men for any discussion, the FGDs of women and men were conducted separately at a time which was convenient to them. All the FGDs were conducted in a private common place in the communities ensuring that the discussion was not interrupted by others. We used the Consolidated Framework for Implementation Research (CFIR) to guide the conceptual design of the study, including the semi-structured interview guide (Supporting information file) [17]. The CFIR provides a meta-theoretical framework of 38 “constructs” that affect implementation under five major domains, including (1) the intervention, (2) the inner setting, (3) the outer settings, (4) the individuals involved, and (5) the process for accomplishing the intervention. However, for this sub-study, we used one of the CFIR constructs, ‘knowledge & beliefs about the intervention’ specifically inquired about community member knowledge, beliefs, and attitudes that shape motivation to participate in cMDA. In addition, ‘STH knowledge’ code was used to understand community members knowledge about STH transmission and prevention. The interview guide was translated into the local language (Tamil) and used by two trained social scientists at India DeWorm3 site, a facilitator and a notetaker, fluent in the local language. The FGDs were recorded on digital voice recorders.

### Data analysis

The audio recorded FGDs were transcribed verbatim in the local language and then translated along with field notes into English. The English transcripts were coded using a mix of *a priori* thematic coding with a codebook informed by 29 of the 37 constructs across all five CFIR domains, 7 non-CFIR constructs, and parsimonious *in vivo* open coding as necessary. Each transcript was coded independently by two coders using a codebook and a largely deductive approach. Concordance between codes was reviewed and resolved during consensus meetings. Coding was conducted with ATLAS.ti 8.0 using a deductive approach.

## Results

A total of 65 adult community members participated in the FGDs, including 33 women and 32 men (Table 1). The age of women who participated in the FGDs ranged from 19–65 years (median 40), most of them were married, and belonged to the Hindu religion. Nearly half (47%) of the women completed middle or higher secondary school. Most of them (79%) were daily wage labourers, had a petty business, or were working on their farms and the rest were homemakers. The age of men who participated in the FGDs ranged from 21–75 years (median 58) of age. Most of them were married and all were Hindus. The majority of males (72%) completed middle or higher secondary school. Most men (59%) were farmers and others were engaged in small business activities or were retirees.

**Table 1:** Background characteristics of the FGD participants.

****Variables****	****Female (n = 33)****		****Male (n = 32)****	
	****Number****	****Percent****	****Number****	****Percent****
Age				
19–30	7	21	3	9
31–50	18	55	9	28
51–75	8	24	20	63
Currently married	32	97	30	94
Education				
No education	6	18	5	16
Primary	12	36	4	13
Middle or higher secondary	15	45	23	72
Occupation				
Home makers	7	21	-	-
Daily wage labourers/farmers	19	58	23	72
Petty business/ retirees/other jobs	7	21	9	28

Broadly, the results presented include knowledge among men and women about STH in general; the awareness of STH among adults; what they believe could prevent STH; and their attitude to cMDA and cMDA strategy they believe would result in high treatment coverage. Overall, women needed more prompts to continue the discussion and answered briefly whereas men were more descriptive in their response.

### There are common misnomers regarding the definition of STH

Men and women across all clusters were aware of STH and other helminth infections but also had some misconceptions. They used various local terms to describe helminths, such as *Kudarpuzhu* (intestinal worm), *Naakupoochi* (whipworm), *Kokkipuzhu* (hookworm), *Naadapuzhu* (tapeworm), *Urundaipuzhu* (roundworm), *Attapoochi* (intestinal leech), *Sirumpuzhu* (small worm), *Arisipuzhu* (rice worm), and *Mannpuzhu* (soil worm). Mostly women described STH by their size, colour, and number using terms such as ‘long’, ‘as large as the finger’, ‘small’, ‘round’, ‘white’, and ‘10–15 worms together’. They were aware that STH were located in the intestine, that they multiplied in the intestine and expelled in the faeces. As they discussed, six women and three men seemed to conflate helminths with earthworms or with other insect larvae that breed in water, including rainwater.

### Stigma regarding helminth infections influences treatment-seeking in adults, highlighting the potential importance of a cMDA approach

Both genders recognized that individuals of any age could be infected with STH. Participants described adults experiencing helminth-associated symptoms such as stomach pain and frequent diarrhoea, as well as expelling helminths after taking purgatives. However, with the exception of one man and one woman, none of the adults would speak about their own experiences with helminth infections and many indicated that helminth infections amongst adults are stigmatized;

*“No one (adults) will reveal in the public about it (worms)*, *sir*. *When the doctor comes*, *many people are asking (for the tablet)*, *they have that worm*.*”* (# 8, Women FGD, Cluster 34)

*“I have lots of intestinal worms… All this time I kept quiet because I did not want to tell here… I get lot of stomach pain… stomach pain stops after taking the tablet*.*”* (# 4, Men FGD, Cluster 34)

### Women are more aware of STH signs and symptoms

Women (15) were aware of more signs and symptoms of STH than men (7). Both genders mentioned stomach pain as the main symptom of STH infection. Men, in addition, stated diarrhoea as a symptom of STH infection while women discussed ‘bloating of the abdomen’, loss of appetite, ‘low blood flow’ indicating anaemia, loss of weight, and pain in hands and legs indicating lethargy. One woman said that helminth infections could incapacitate adults to an extent that it could prevent them from doing any work.

*“They will not go anywhere outside; they will remain in their houses; they will not be able to do any work*.*”* (# 8, Women FGD, Cluster 12)

Although participants narrated many ill-effects of STH infections, one man believed that intestinal helminths were helpful and necessary for digestion. He said,

*“This worm will eat that food… it should be there only then you can digest… without the worm*, *it will not digest*.*”* (# 2, Men FGD, Cluster 17)

### Both genders were aware that poor hygiene practices and sanitation affect STH transmission

Participants of both genders, eight women and three men, across all clusters discussed that open defecation, not wearing footwear, and eating and drinking without washing hands increased the risk of STH transmission. Two men in two of the clusters noted that male agriculture workers typically defecate in the open because they cannot go home to use the toilet. As a result, they believed that farmers are more likely to have helminth infections as they tended to handle the soil and may not practice proper hand hygiene. They discussed as,

*“Sir*, *in the village most go out for open defecation*. *Even if there is a toilet in the house*, *when they go for work in the field*, *they also go to (defaecate)… it may not possible to come back home for it*, *because they leave at 4 or 5 in the morning… so that also why they go to the latrine in the field*.*”* (# 3, Men FGD, Cluster 12)

*“People who toil in the land may not have clean hands*, *so they may not be without worm infection*. *All will have*, *all who live in the villages will have*.*”* (# 6, Men FGD, Cluster 17)

One of the men described that helminth infections are associated with walking barefoot on the soil where people had defecated as,

*“As far as what we have heard… when the worm is passed through the faeces in the open place*, *it stays there for 90 days… when we stamp that with our feet… if someone else stamps that… there are chances for it to spread through our feet*.*”* (# 1, Men FGD, Cluster 34)

### Women recognized that safe sanitation plays an important role in prevention of STH transmission along with treatment

Six women discussed that washing hands with soap after defecation, keeping their nails cut and clean, using the toilet for defecation, drinking boiled water, and wearing footwear while going to the toilet or anywhere outside of the house would help in the prevention of STH transmission. They discussed as,

*“We should drink boiled water*, *should wash hands*, *after defecation first thing is to wash hands with soap… nails should be cut neatly… for prevent spreading of diseases*, *mainly you should cut nails and keep it clean*.*”* (# 11, Women FGD, Cluster 12)

*“When we go to the bathroom*, *we should wear slippers and go*, *should not defecate in the open*, *should go only in a toilet*.*”* (# 2, Women FGD, Cluster 15)

### Both genders had a positive attitude towards cMDA

Both men (15) and women (23) across all clusters believed that deworming tablets will help treat and prevent STH infections. They were aware of the benefit of STH treatment that children received from schools and believed that cMDA would help prevent new STH infections in children that might be acquired from parents. The men believed that multiple rounds of treatment may be required to eliminate STH, as they discussed,

*“If we give to everyone at the same time*, *it will get controlled at the same time… if you see*, *because it is given only to the children it spreads from the adults… from parents to the children*. *So*, *if we give to everyone at the same time*, *there is a possibility of it to come down*.*”* (# 2, Men FGD, Cluster 34)

*“This is possible only if everyone cooperates*. *If I eat and he does not eat then how it will be destroyed*, *all should be united… all should cooperate in this project*, *only then we can destroy…*. *surely*, *we can eradicate*.*”* (# 8, Men FGD, Cluster 17)

*“Surely*, *we can eliminate this if all will eat the tablet… we can cut about three-fourths in six months*, *in the next six months we can do everything*, *we can solve all the problems if it is done two to three times… suppose there are ten houses in our street… two households may have gone for a marriage*.*”* (# 3, Men FGD, Cluster 15)

Women knew that deworming medication will reduce worm load but gradually indicating, many rounds of treatment would be required to control STH. They said,

*“By giving this (tablet)*, *a high level (infection) can be reduced to some extent*. *Now in the beginning… it will reduce only a little and if you keep giving it for a year*, *there is a possibility for it to reduce… regularly taking tablet it will reduce little by little*, *automatically*.*”* (# 5, Women FGD, Cluster 17)

### Men and women had different concerns regarding cMDA for STH

Men and women had different concerns regarding the drug distributed in cMDA for STH infections. One woman and four men from three clusters discussed that if it is given free everyone will accept but one woman said that some people may also have reservations about the quality of the tablet that is distributed free of cost. They said,

*“They will doubt whether this tablet is original or adulterated… they might think like why they are coming and giving the free tablet for intestinal worms*. *Giving money and getting is a different thing… they are coming in search of our house and giving the tablet for intestinal worms for free… should we eat this or not*?” (# 5, Women FGD, Cluster 17)

Women (17) across all clusters and men (7) in two clusters discussed the fear of side effects of the deworming tablet such as vomiting, diarrhoea, fever, drowsiness, and dizziness as a driver of low compliance. Men expressed concerns over potential contraindications of deworming tablet with medications for hypertension and diabetes, or allergies as,

*“Issues will come*, *will get drowsy*, *will vomit*, *will get diarrhea… so*, *in fear*, *we may eat or just leave it without eating… we will have a fear about what will happen… you will give the tablet and leave*, *then we get diarrhea and we get vomiting… we will have come in search of you… how can we search and find you*?*”* (# 6, Women FGD, Cluster 15)

*“They may doubt if they will have any side effects… will there be any side effect by eating this tablet… whether any other disease will come… whether people who already have low BP and sugar and those who are regularly eating tablets can eat this tablet*.*”* (# 1, Men FGD, Cluster 15)

Men reported that adult compliance will be driven by perceptions of infection risk and individuals who don’t think they are infected will not consume the tablet;

*“Those who do not have stomach pain and those who do not have an ulcer*, *they will know that they do not have the worm… if they do not have any of these three symptoms*, *it means that they do not have worms… because of that*, *they will not take it*.*”* (# 7, Men FGD, Cluster 34)

Although many concerns were raised about drug safety, men commented that the government is already giving tablets to children, and thus they are likely safe for adults during cMDA as well.

*“If it is (tablet) fine with children it will be fine for adults also*. *That is the proof*. *They gave to children and they are fine*, *so adults will also be fine*, *it is proof that we gave to children and they are fine*.*”* (# 2, Men FGD, Cluster 15)

### Men and women believed that community sensitization and door to door drug delivery will facilitate acceptance to cMDA

Both men and women across all clusters said that door to door drug delivery is preferable to other delivery methods, such as fixed-point delivery. Additionally, men believed that misconceptions and barriers to cMDA could be overcome if the benefits and safety of the drug are clearly explained by trustworthy persons like government health care providers, and that will help in building people’s confidence.

*“When that explanation is given*, *people will take and eat it*. *With this tablet these intestinal worms will not come*, *these problems will not arise*, *if at all there is any worm it will automatically come out when you pass stool if you eat this tablet*. *If we say all this*, *they will take and eat… if one person goes house to house*, *they will be familiar and they will know this person is giving for this”* (# 6, Men FGD, Cluster 12)

*“If you give door to door then… its benefits will be known to all*, *only then the people will have the confidence to take it… so first they should do door delivery*.*”* (# 4, Men FGD, Cluster 17)

“*In the school means*, *they will give only to the children*. *If you give in the house and if you give to all members that will be good*.*”* (# 1, Women FGD, Cluster 34)

Men also alerted that community-wide deworming could be limited because some would have gone out for work or would be absent at the time of cMDA;

*“… if you give to 100 persons*, *30 or 25 persons may not get it… they would have gone out*, *some persons would have been absent*, *or would have forgotten and left it…”* (# 3, Men FGD, Cluster 15)

## Discussion

This study describes gendered perspectives of knowledge, beliefs, and attitudes regarding STH transmission and cMDA. Barriers to acceptance and utilization of health services can be addressed if community perspectives, including gender specific perspectives, are considered during program planning and implementation. Analysis of national-level datasets shows that although overall gender disparity is improving over time among children’s health and care-seeking behaviours like immunization, stunting, and treatment-seeking for acute respiratory infections, but it persists probably due to gender discrimination favouring the male child [18]. Among adults, reproductive health and HIV related programs have shown improvement in gender and health inequity by enhancing participant’s agency to adopt a behaviour [19]. In Indonesia, women have been shown to be more likely than men to have received MDA for LF, likely because the MDA was conducted during the day when men were not available. However, in this study, women were less likely to have consumed the drug, due to avoidance of MDA among pregnant and lactating mothers [20]. Compliance by gender in MDA programmes, including LF programmes, varies across states in India. Reasons for non-compliance include fear of side effects, forgetting to consume, too many tablets, and no repeat visit to houses; however, to date, drivers of differential non-compliance by gender including household power relations influencing participation have not been systematically reported [15,16]. Understanding gender-specific factors influencing demand for MDA and treatment uptake is important in order to maximize program design and implementation.

Men and women in this study demonstrated similarly high levels of knowledge regarding STH and STH transmission probably because of government’s biannual school deworming program. The high level of knowledge about STH infections observed in this study may be due to the intensive national deworming day and WASH government programming and sensitization in this study area. Female participants appeared to have a more comprehensive understanding of the signs and symptoms associated with STH infection. This is possibly because women have more opportunities to learn about maternal, child health, and adolescent health programs where deworming could have been discussed in the relation to nutrition, anaemia, and growth. Both men and women were aware that STH infections could affect adults and were not limited to children alone, and they believed that adults may be re-infecting children after they are treated as part of the national deworming day. Both men and women knew that STH transmission is associated with inadequate hygiene and sanitation. Gender norms, as also reported in other studies [11], seems to shape vulnerability to STH infections as women highlighted transmission risks related to sanitation and food consumption while men associated their risk to STH infection with farming because it is not feasible while farming to go home to use a toilet. They believed that loss of appetite and loss of weight due to STH infections made infected individuals prone to many other illnesses. However, this study also showed that misconceptions about having worms may also prevail in the community, including the belief that worms aid in digestion though the source of this misconception is not known. This is similar to findings reported in a study from Bangladesh [21]. Studies comparing knowledge of STH among men and women in India are limited. Available data do suggest that knowledge of LF is similar among men and women, while knowledge of tuberculosis and HIV appears higher among men than women [16,22,23].

Our study showed that stigma of having STH infections was probably a barrier to accessing treatment by the adults and therefore, both men and women felt that cMDA might alleviate such concerns regarding STH stigma, which otherwise prevented adults from openly seeking treatment. Systematic reviews have shown stigma resulting in feeling of shame and social exclusion to be a barrier to accessing treatment for other NTDs like LF and leprosy [24,25]. Our study suggests that stigma may be an important driver of non-compliance with NTD programs even for relatively asymptomatic infections. This finding supports the likelihood that cMDA for STH may be a preferred approach to increasing access of STH chemotherapy in adults, as this approach avoids discrimination in drug delivery between those infected and not infected.

While both men and women had concerns about the safety of deworming medications, women were more concerned about drug side-effects and drug quality than men. Fear of side effects may be due to personal experience of side-effects or from reports from other community members experiences of MDA programmes, including those for STH, LF, and other NTDs. [15]. Women, influenced by gender roles, may be particularly concerned about side-effects, in themselves or their family members, because as the primary caregivers they are perceived to be responsible for family health and welfare. Also, observing side-effects among family members and neighbours can create fear, and lead to non-compliance.

As caregivers of the family, women discussed that only children benefit from school deworming, but with cMDA, everyone in the household. Men, on the other hand, generalized as cMDA will benefit everyone. The men and women in this study believed that door-to-door drug distribution by trusted community care providers would help in community participation in the cMDA programme. As men described, door to door distribution will also provide opportunities for information sharing at the household level through interpersonal communication, provide an opportunity to clarify concerns and overcome fear and misgivings, and establish trust with the drug distributors, and thus facilitate high coverage. Importantly, as noted by men in this study, the timing of drug distribution in the community should be considered during cMDA micro-planning, as many would be out of home for work during the day and some would be temporarily migrated for work or other commitments. Studies have shown that the availability of community members at home during MDA can be as low as 50% [15].

Unlike other studies, female participants in our study did not express that males have all decision-making control regarding household participation in cMDA. Studies from Indonesia and Ethiopia indicated that women’s participation in MDAs was controlled by men in their families [13,26]. One study in Uganda found that women indirectly had greater decision-making power to participate in NTD treatment programmes because men were away for work and were absent during MDAs [27].

We used the CFIR framework to structure our DeWorm3 qualitative data collection and analysis, from which this sub-study was conducted. The CFIR is a determinants framework, used to identify facilitator and barriers to implementation. However, the CFIR does not incorporate gender theory, and one of the CFIR constructs, ‘knowledge & beliefs about the intervention’, emerges as salient drivers of thematic content. This construct explores individuals’ attitudes towards, and value placed on the innovation, as well as familiarity with facts, truths, and principles related to the innovation. Although the CFIR does not include gender theory, these constructs drove key findings that mirror constructs included in other gender-based theories such as Harvard Analytical Framework and Women’s Empowerment Framework [28,29]. These gender-based theories include constructs that evaluate factors influencing differences in access to services by women and men and control of resources.

A limitation of this study is that the FGDs were not mixed gender, which might have allowed us to understand obvert agreements and disagreements between men and women regarding perceptions of STH programming. However, FGDs were conducted separately in order to ensure safe opportunities for women to speak freely. There is also a possibility that gender relations within the households that were approached could have influenced the participation of community members, particularly of women, in the FGDs. The study design limited participation of only one person from a household, and it cannot be assumed that the perceptions of the selected individuals are representative of their household or community. Moreover, community members of all ages were included in FGDs which limited deeper understanding of the intersectional perceptions of cMDA, influenced by both age and gender. Further, we did not use a gender analysis framework to design and analyse the data from these FGDs. This study discusses cMDA for STH and did not compare it with delivery of school-based deworming which is the standard of care.

These findings suggest, that (i) both men and women should be informed about the rationale for multiple rounds of cMDA for STH regardless of STH infection status to achieve sustained high compliance; (ii) women in particular, need to be informed that the drug distributed in cMDA is safe and it is the same drug that is distributed to children in schools by the government; (iii) though women are more concerned about side-effects of the treatment, both men and women should have or know the contact details of the person/s to call in the event of any adverse events; (iv) misconceptions raised by some men like STH infections aiding in digestion should be clarified, while the knowledge the women had about effects of STH infections on health should be reinforced; (v) behaviour change to adopt WASH practices should be encouraged; and (vi) information about cMDA and drug delivery should be carried out by trusted government frontline health workers.

## Conclusion

This study showed high baseline knowledge of STH infections among men and women residing in this community, indicating fairly equitable access to information about these infections. Men and women had similar positive beliefs and attitudes towards cMDA for STH, indicating broad community willingness to participate. Some gender differences were observed, particularly with women expressing concerns regarding drug quality and men expressing concerns that the timing of cMDA delivery to the household may affect the likelihood of reaching migratory individuals or those away from the household during the day. This study identified some general and gender-specific key messages that should be incorporated within communication strategies for future cMDA programmes, including the rationale for cMDA, information on drug safety and information, and details of whom to contact in case of adverse events.

## Supporting information

S1 Text Formative Qualitative Research Question Guide for STH-cMDA, DeWorm3 study. (PDF)
