# Supplementary material for: Gender differences in the perceived need for community-wide deworming: Formative qualitative research from the DeWorm3 study, India
Source: PLoS Negl Trop Dis. 2020 Nov 25;14(11):e0008829. doi: 10.1371/journal.pntd.0008829 (PMC7688162; doi:10.1371/journal.pntd.0008829)
Supplement: S1 Text — (PDF) [file pntd.0008829.s001.pdf]

DeWorm3 Qualitative Research Interview Guide
Focus Group Discussions

Stakeholder Level: Community Members **Study Time Point:** Baseline

Facilitator Instructions:

Please familiarize yourself with the questions below prior to consenting the focus group participant. Review **SOP 804** for information on how to consent participants and **SOP 805** on how to facilitate the focus group, manage the audio recorder, and other key details.

Once you have consented the participant(s) and turned the audio recorder on. Please welcome the individual, thank them for their participation, and make them feel comfortable. Try asking a few questions to help them relax e.g. questions about where they are from or their work.

Please use the question guide below and ensure that all questions are answered. If answers are brief or not sufficient to answer the question asked, some follow-up question prompts have been provided in italics.

Please take notes as appropriate if there are important details that you want to follow-up on, or if you want to note key details about the individual's behavior, attitude, or reactions to questions.

#	Question
**1**	☐ What advantages does community-wide MDA for STH have compared to school-age targeted programs? What disadvantages?
**2**	☐ When delivering community-wide MDA for STH, what local adaptations should be made so that the intervention is effective?<br>☐ What aspects of the intervention should not be adapted?
**3**	☐ What barriers might community members face in participating in community-wide MDA for STH?<br>*Follow-up question:*<br>*What could be done to overcome these barriers?*
**4**	☐ Do you remember the lymphatic filariasis (LF) MDA programs that used to occur in this area? What do you remember about them? Did you participate in those treatment days by swallowing the medicines given to you? Why or why not? Has that affected your interest in participating in the current mass treatment days?
**5**	☐ Do you think that community-wide MDA could interrupt STH transmission (i.e. stop the spread of intestinal worms) in your setting? Why or why not?
**6**	☐ Who are the key influential individuals or organizations to get buy-in from during the DeWorm3 study?
**7**	☐ How should community members be informed about community-wide MDA or other DeWorm3 interventions before they occur?
**8**	☐ What are some strategies to achieve high MDA treatment coverage?
